# Reversed Halo Sign in Tuberous Sclerosis Complex

**DOI:** 10.1155/2013/428501

**Published:** 2013-09-15

**Authors:** Kazuhiro Suzuki, Kuniaki Seyama, Takuo Hayashi, Yuki Yamashiro, Akihiko Shiraishi, Ryohei Kuwatsuru

**Affiliations:** ^1^Department of Radiology, Faculty of Medicine, Juntendo University, 2-1-1 Hongo, Bunkyo-ku, Tokyo 113-8421, Japan; ^2^Department of Respiratory Medicine, Faculty of Medicine, Juntendo University, 2-1-1 Hongo, Bunkyo-ku, Tokyo 113-8421, Japan; ^3^Department of Human Pathology, Faculty of Medicine, Juntendo University, 2-1-1 Hongo, Bunkyo-ku, Tokyo 113-8421, Japan

## Abstract

We describe a reversed halo sign in a teenage girl with tuberous sclerosis complex (TSC). Lung manifestations of TSC include lung cysts corresponding to lymphangioleiomyomatosis and small nodules indicating multifocal micronodular pneumocyte hyperplasia (MMPH). However, a reversed halo sign in TSC has never been reported. The lesion was microscopically consistent with MMPH. Immunohistological findings also supported the notion that the lesion is associated with TSC.

## 1. Introduction

Tuberous sclerosis complex (TSC) is an autosomal dominant inherited neurocutaneous syndrome characterized by various hamartomatous lesions in various organs [1]. Pulmonary manifestations of TSC include lung cysts corresponding to lymphangioleiomyomatosis (LAM) and small nodules indicating multifocal micronodular pneumocyte hyperplasia (MMPH). Pulmonary manifestations occur in 1%–2.3% of patients with TSC, but recent reports indicate that pulmonary LAM can be radiologically detected in 26%–39% of female patients with TSC [1, 2]. MMPH has been considered a rare manifestation of TSC. However, Muzykewicz et al. described that 58% of patients with TSC have pulmonary nodules that represent MMPH [3]. Typical radiological features of MMPH are multiple tiny nodules that are diffusely and randomly scattered throughout the lung [3, 4]. Kobashi et al. noted that computed tomography (CT) identified ground-glass opacity (GGO) in all of the 15 patients with MMPH [5]. On the other hand, Muzykewicz et al. described that 67% of TSC patients with multiple nodules had both solid and ground-glass nodules.

The reversed halo sign is defined as focal rounded areas of GGO surrounded by a more or less complete ring of consolidation that can be visualized by CT [6]. This sign was initially considered specific to cryptogenic organizing pneumonia [7]. However, the reversed halo sign has been associated with various infectious and noninfectious clinical entities [8].

Here, we describe our experience of a unique Japanese patient with TSC and the reversed halo sign.

## 2. Case Report

**Figure 1:**
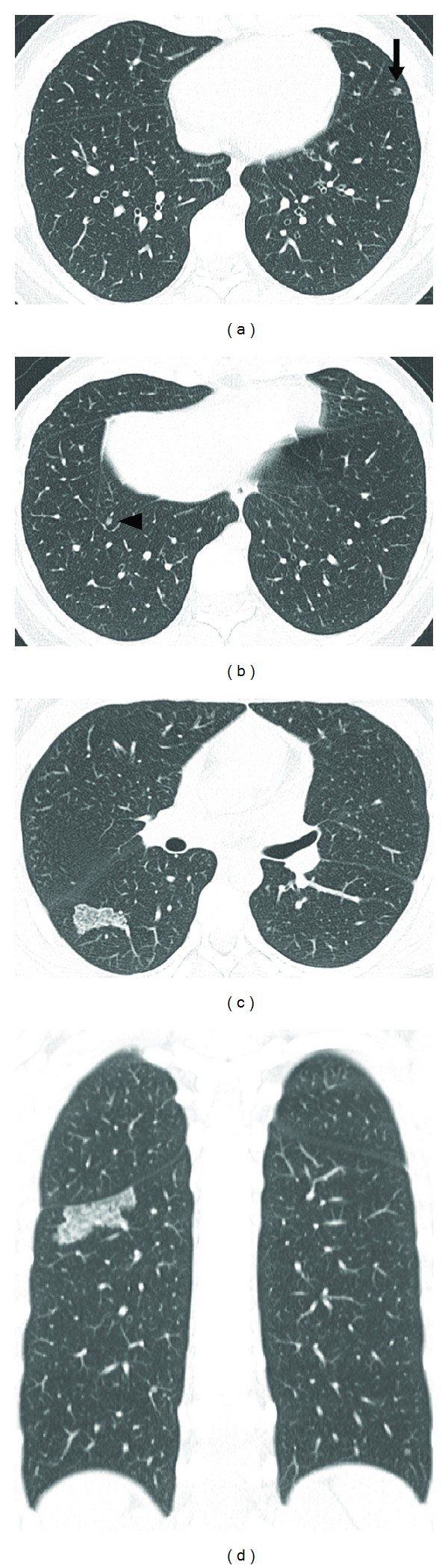
Chest CT findings. (a), (b) Axial CT images (1.5 mm thickness) of the chest show small nodules with ground-glass opacity in the lingular segment of the left upper lobe ((a), arrow) and the lateral segment of the right lower lobe ((b), arrowhead). (c) Axial CT image (1.5 mm thickness) shows ground-glass opacity with 30 mm diameter surrounded by dense linear consolidation (reversed halo sign) in the superior segment of the right lower lobe. (d) Coronal reconstructed image (3 mm thickness) also shows revered halo sign in the superior segment of the right lower lobe.

**Figure 2:**
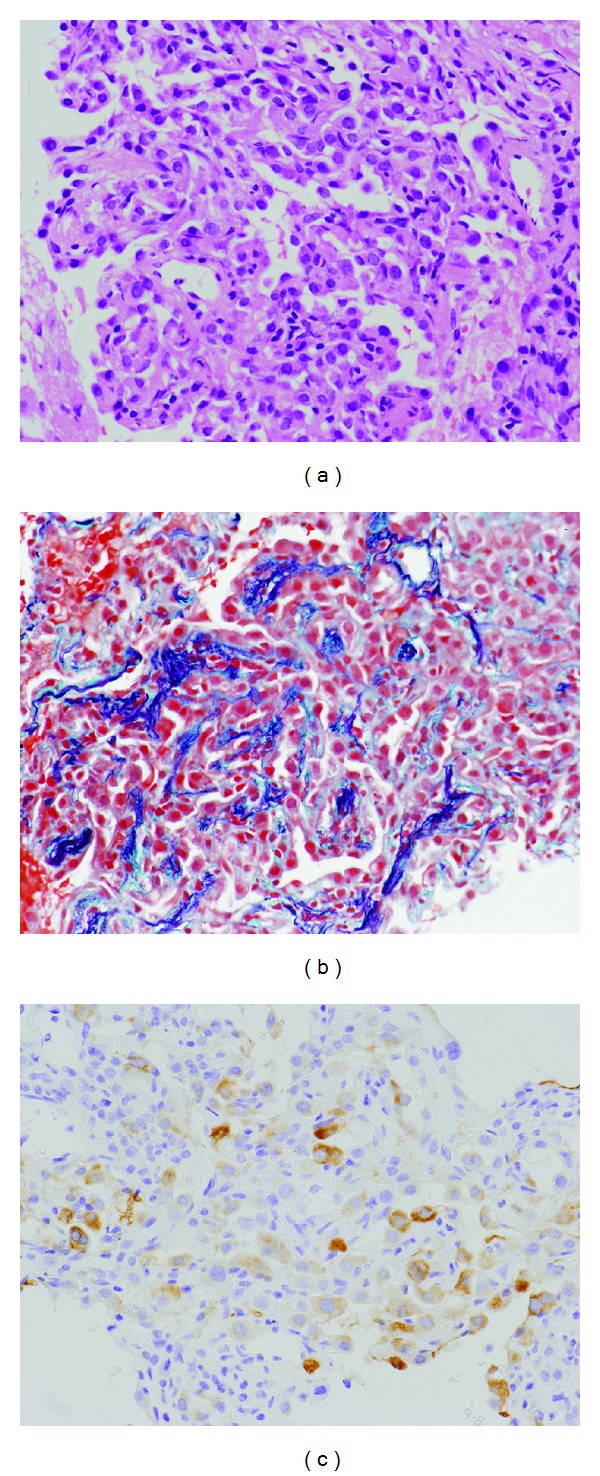
Microscopic findings. (a) Enlarged cuboidal cells have abundant, eosinophilic cytoplasm and large, round nuclei lining mildly thickened alveolar septa (hematoxylin and eosin stain). (b) Alveolar septa are focally composed of thickened elastic fibers (Elastica-Masson trichrome stain). (c) Enlarged cuboidal cells are positive for phospho-S6 ribosomal protein.

A teenage girl was referred to our institution with abnormal findings on chest CT and TSC diagnosed by a dermatologist based on skin lesions. She was asymptomatic upon admission, and laboratory data were normal. Chest CT showed multiple nodules in both lungs (Figures 1(a) and 1(b)) and GGO with a 30 mm diameter surrounded by dense linear consolidation in the superior segment of the right lower lobe (Figures 1(c) and 1(d)). Since pulmonary artery and vein passed the lesion showing GGO and did not correspond with surrounding linear consolidation, the lesion agreed with the reversed halo sign. No lung cysts suggested LAM, and CT revealed that she was free of liver and kidney diseases suggesting angiomyolipoma. Multiple lung nodules were diagnosed as MMPH because the patient had a background of TSC and some had GGO [2, 3, 5]. The lesion with reversed halo sign was difficult to diagnose. Differential diagnoses include cryptogenic organized pneumonia [7, 9], infectious lung disease [8], and atypical MMPH. Because the shape and size of the lesion had not changed on CT images by 3 months later, a diagnosis could not be concluded based on imaging findings and the clinical course. Pathological confirmation was required to determine the optimal therapeutic strategy. The patient provided written informed consent to undergo a CT-guided transthoracic needle biopsy of the lesion, which proceeded uneventfully, and she was discharged a few days later. Histopathologically, enlarged cuboidal cells with abundant, eosinophilic cytoplasm, and large, round nuclei, lined a mildly thickened alveolar septa. The airspaces of the lesion were filled with these cells and alveolar macrophages. The alveolar septa focally comprised thickened elastic fibers and lymphocytic infiltration (Figures 2(a) and 2(b)). The enlarged cuboidal cells were immunohistochemically positive for phospho-S6 ribosomal protein (p-S6), a downstream protein of the mammalian target of rapamycin (mTOR) signaling pathway that is regulated by the *TSC* genes (Figure 2(c)). The histological and immunohistochemical findings were consistent with MMPH.

## 3. Discussion

We encountered MMPH with typical, tiny, and fine nodules together with the reversed halo sign on CT images of a young patient with TSC. Furthermore, the 30 mm GGO responsible for the reversed halo sign seems the largest reported as MMPH. To the best of our knowledge, a reversed halo sign has never been identified in TSC, and two different coexisting features representing MMPH is an extremely rare lung manifestation of TSC. Generally, pulmonary involvement of TSC is known as LAM and MMPH. The latter is histologically characterized by a multicentric, well-demarcated nodular growth of bland-looking type II pneumocytes along an alveolar septa that exhibits fibrous thickening, increased numbers of elastic fibers, and aggregated alveolar macrophages [10]. The radiological features of MMPH are multiple tiny nodules that are randomly and diffusely scattered throughout the lung [4]. Muzykewicz et al. recently described CT findings of either solid or ground-glass MMPH nodules with diameters ranging from 2 to 14 mm that are diffusely distributed in the lungs of patients with TSC [3]. No single nodule has presented with both solid and ground-glass features [3]. Two studies have identified MMPH nodules of <20 mm [3, 5]. An analysis of MMPH identified one Japanese patient with TSC and atypical large nodules (20 mm) and infiltrative shadows, but coexisting small lesions were not described [5]. Muzykewicz et al. also reported a target-like appearance, with ground-glass nodules of increasing density around the periphery in 5 of 42 patients with TSC [3]. However, their report includes images that are not typical reversed halo signs and does not mention coexisting nodules of various sizes and opacity in these patients.

The reversed halo sign in our patient was histologically consistent with MMPH, and the immunohistochemical positivity for p-S6 protein supported a diagnosis of MMPH. The histopathological findings of the reversed halo sign in cryptogenic organizing pneumonia are considered to arise from the central GGO corresponding to an area of alveolar septal inflammation and cellular debris in the alveolar spaces, whereas ring-shaped or crescentic peripheral air-space consolidation corresponds to areas of organizing pneumonia within the distal air spaces [9]. The appearance of the lesion on CT could not be precisely correlated with the histopathological findings because the specimen obtained by needle biopsy was only part of the entire lesion. Apart from pneumocytic hyperplasia, an inflammatory process was identified in the specimen. However, we could not precisely correlate these histopathological findings with GGO and the surrounding dense linear consolidation that are two constituents of the reversed halo sign in CT.

In summary, we described a patient with the reversed halo sign in TSC that was microscopically consistent with MMPH. Two different radiological features arising from one pathological process would be quite challenging for radiologists to precisely diagnose. Nonetheless, those atypical radiological features such as large GGO and a reversed halo sign might indicate that MMPH should be borne in mind.
